# Supplementary material for: Content-rich biological network constructed by mining PubMed abstracts
Source: BMC Bioinformatics. 2004 Oct 8;5:147. doi: 10.1186/1471-2105-5-147 (PMC528731; doi:10.1186/1471-2105-5-147)
Supplement: Additional File 2 — The original results of the above study (non-essential files are deleted to keep the file size under the limit set by BMC bioinformatics). [file 1471-2105-5-147-S2.bz2 › chilibotAdditionalFile2/dip30/643ID11106733E11030/html/HSLU_HSLV.html]

 


 **HSLU** and **HSLV** 
  
Found 35 abstracts in PubMed, retrieved 30.  
 

 What does Google say? 
 PDF only 
| .edu only 

---

**Interactive relationship** (e.g. stimulation, inhibition, etc)

**Stimulatory relationship**- The Escherichia coli and T. maritima  **HslU**  proteins mutually stimulate  **HslV**  proteins from both sources, suggesting a conserved activation mechanism.  Ref: 12646382 Biophys Chem, 2003
- Histone that could stimulate the ATPase activity of  **HslU**  also increased the rate of the ATP dependent peptide hydrolysis by  **HslV** , although to a much lesser extent than by poly L lysine.  Ref: 8954932 Biochem Biophys Res Commun, 1996
- Thus, the poly L lysine mediated increase in the ATPase activity of  **HslU**  appears to be responsible for the dramatic activation of the ATP dependent peptide hydrolysis by  **HslV** .  Ref: 8954932 Biochem Biophys Res Commun, 1996
- Thus,  **HslU**  appears to have two antagonistic functions one as a chaperone for promotion of the ability of SulA in cell growth inhibition by preventing SulA aggregation and the other as the regulatory component for elimination of SulA by supporting the  **HslV**  mediated degradation.  Ref: 10908725 FEBS Lett, 2000
**Neutral relationship**- The hexameric  **HslU**  ATPase binds the dodecameric  **HslV**  peptidase and forms an ATP dependent HslVU protease.  Ref: 11709174 Structure Camb , 2001
- In the crystalline complex, the I domains of  **HslU**  interact with  **HslV** .  Ref: 11114186 Proc Natl Acad Sci U S A, 2000
- Two hexameric ATP binding rings of  **HslU**  bind intimately to opposite sides of the  **HslV**  protease.  Ref: 11106733 Cell, 2000
- RESULTS We present a 3.0 A resolution crystal structure of HslVU with an  **HslU**  hexamer bound at one end of an  **HslV**  dodecamer.  Ref: 11250202 Structure Camb < MedlineTA>Structure Camb , 2000
- A third, mostly alpha helical domain in  **HslU**  mediates the contact with  **HslV**  and may be the structural equivalent of the amino terminal domains in proteasomal AAA ATPases.  Ref: 10693812 Nature, 2000
- The binding of one  **HslU**  hexamer to one end of an  **HslV**  dodecamer in the 3.0 A resolution structure opens both  **HslV**  central pores and induces asymmetric changes in  **HslV** .  Ref: 11250202 Structure Camb < MedlineTA>Structure Camb , 2000
- The C terminal tails of  **HslU**  ATPase act as a molecular switch for activation of  **HslV**  peptidase.  Ref: 12011053 J Biol Chem, 2002
- It is known from other work that when the  **HslU**  hexamer binds its cognate protease  **HslV** , the carboxy terminal helices of  **HslU**  protomers distend and bind between  **HslV**  subunits.  Ref: 11468391 Acta Crystallogr D Biol Crystallogr, 2001
- In the prokaryotic homolog of the eukaryotic proteasome, HslUV, the double donut  **HslV**  protease is allosterically activated by  **HslU** , an AAA protein of the Clp Hsp100 family consisting of three amino terminal, carboxy terminal, and intermediate domains.  Ref: 12823960 J Mol Biol, 2003
- HslVU is a bacterial homolog of the proteasome, where  **HslV**  is the protease that is activated by  **HslU** , an ATPase and chaperone.  Ref: 12032294 Proc Natl Acad Sci U S A, 2002
- and the structure has been solved to 2.5A resolution, revealing an assembly in which a  **HslU**  DeltaI hexamer binds one end of the  **HslV**  dodecamer.  Ref: 12823960 J Mol Biol, 2003
- When the complex forms, the carboxy terminal helices of  **HslU**  distend and bind between subunits of  **HslV** , and the apical helices of  **HslV**  shift substantially, transmitting a conformational change to the active site region of the protease.  Ref: 11106733 Cell, 2000
- The conformation of the protomers of the  **HslU**  DeltaI complexed  **HslV**  hexamer is similar to that in the symmetric wild type HslUV complex, while the protomer conformation of the uncomplexed  **HslV**  hexamer is similar to that of  **HslV**  alone.  Ref: 12823960 J Mol Biol, 2003
- On the basis of the structure of a HslUV complex, a mechanism of allosteric activation of the  **HslV**  protease, wherein binding of the  **HslU**  chaperone propagates a conformational change to the active site cleft of the protease, has been proposed.  Ref: 12054822 J Mol Biol, 2002
- Here we show that the C terminal tails of  **HslU**  play a critical role in the interaction with and activation of  **HslV**  peptidase.  Ref: 12011053 J Biol Chem, 2002
- Here we report site directed mutagenesis, peptide activation, and fluorescence experiments that strongly support the functional relevance of the C terminus insertion mechanism we find that mutations in  **HslV**  that disrupt the interaction with the C termini of  **HslU**  invariably lead to inactive enzyme.  Ref: 12032294 Proc Natl Acad Sci U S A, 2002
- A synthetic tail peptide of 10 amino acids could replace  **HslU**  in supporting the  **HslV**  mediated hydrolysis of unfolded polypeptide substrates such as alpha casein, as well as of small peptides, suggesting that the  **HslU**  C terminus is involved in the opening of the  **HslV**  pore for substrate entry.  Ref: 12011053 J Biol Chem, 2002
- Conversely, synthetic peptides derived from the C terminus of  **HslU**  bind to  **HslV**  with 10 5 M affinity and can functionally replace full  **HslU**  particles for both peptide and casein degradation but fail to support degradation of a folded substrate.  Ref: 12032294 Proc Natl Acad Sci U S A, 2002
- Functional interactions of  **HslV**  ClpQ with the ATPase  **HslU**  ClpY.  Ref: 12032294 Proc Natl Acad Sci U S A, 2002
- These results suggest that, in the reconstituted HslVU complex, the peptide hydrolysis by  **HslV**  occurs in a tightly coupled process with the cleavage of ATP by  **HslU** .  Ref: 8954932 Biochem Biophys Res Commun, 1996
- Whereas  **HslU**  C261V could hydrolyze ATP and support the ATP dependent proteolytic activity of  **HslV**  as well as the wild type  **HslU** ,  **HslU**  C287V could not hydrolyze ATP.  Ref: 9722513 J Biol Chem, 1998
- These results suggest that the  **HslU**  C terminal tails act as a molecular switch for the assembly of HslVU complex and the activation of  **HslV**  peptidase.  Ref: 12011053 J Biol Chem, 2002
- Forty three nucleotides downstream from the  **hslV**  stop codon was found a second heat shock gene  **hslU**  with an ORF of 1401 bp encoding a 53.139 kD polypeptide of 467 amino acids with 49 56% identity to the  **HslU**  homologs from other bacteria.  Ref: 11685516 Curr Microbiol, 2001
- The mutational studies also revealed that both Thr, Ser103, and Ser172, but not Ser124, are involved in the interaction of  **HslV**  with  **HslU**  and hence in the activation of  **HslU**  ATPase as well as in the HslVU complex formation.  Ref: 9257689 FEBS Lett, 1997
- With AMP PNP present, maximal proteolytic activity is observed with a molar ratio of  **HslU**  to  **HslV**  subunits of 1 1, and negative staining electron microscopy shows that  **HslV**  and HsIU form cylindrical four ring structures in which the HsIV dodecamer is flanked at each end by a  **HslU**  ring.  Ref: 9033594 Nat Struct Biol, 1997
- This delay decreased at higher temperatures and with higher  **HslU**  or  **HslV**  concentrations and was eliminated by preincubation of  **HslU**  and  **HslV**  together.  Ref: 9261150 J Biol Chem, 1997

**Non-interactive relationship** (e.g. studied together, co-existance, homology, etc.)

- Further characterisation of pHL1 revealed that resistance to nitrofurantoin was due to the overexpression of the  **hslV**   **hslU**  operon which encodes an ATP dependent protease complex in E. coli.  Ref: 9315728 FEBS Lett, 1997
- HslUV is a prokaryotic proteasome composed of the  **HslV**  protease and the  **HslU**  ATPase, a chaperone of the Clp Hsp100 family.  Ref: 11106733 Cell, 2000
- PCR genome walking identifies a genetic locus comprising two heat shock genes  **hslV**  and  **hslU**  from Leptospira borgpetersenii serovar hardjobovis.  Ref: 11685516 Curr Microbiol, 2001
- We have used electron microscopy and image analysis to examine the structural organization of  **HslV**  and  **HslU**  homo oligomers and the active HslVU enzyme.  Ref: 9033594 Nat Struct Biol, 1997
- HslVU is a two component ATP dependent protease composed of the proteasome related peptidase  **HslV**  beta subunit and the ATPase  **HslU** .  Ref: 9165063 Biol Chem< MedlineTA>Biol Chem, 1997
- These results confirm that  **HslV**  can be activated by binding of a hexameric  **HslU**  DeltaI 6 ring lacking the I domains, that activation is effected through a conformational change in  **HslV**  rather than through alteration of the size of the entry channel into the protease catalytic cavity,.  Ref: 12823960 J Mol Biol, 2003
- Nevertheless,  **HslU**  C287V could support the  **HslV**  mediated proteolysis by forming the HslVU complex in the presence of ATP but not its absence, indicating that ATP binding but not its hydrolysis is essential for proteolysis.  Ref: 9722513 J Biol Chem, 1998
- The bacterial heat shock locus  **HslU**  ATPase and  **HslV**  peptidase together form an ATP dependent HslVU protease.  Ref: 11469873 J Struct Biol, 2001
- Instead, there is conformational flexibility and domain motion in  **HslU**  and a localized order disorder transition in  **HslV** .  Ref: 10693812 Nature, 2000
- HslVU is a two component ATP dependent protease, consisting of  **HslV**  peptidase and  **HslU**  ATPase.  Ref: 11179218 EMBO J, 2001
- Consistent with three electron microscopic studies and one small angle X ray scattering study, three crystal structures show that the nucleotide binding domains of  **HslU**  bind to  **HslV** .  Ref: 11469873 J Struct Biol, 2001
- Here we show that  **HslU**  plays a role not only as a regulatory component for the  **HslV**  mediated proteolysis but also as a molecular chaperone.  Ref: 10908725 FEBS Lett, 2000
- HslVU is an ATP dependent protease consisting of two multimeric components the  **HslU**  ATPase and the  **HslV**  peptidase.  Ref: 10908725 FEBS Lett, 2000
- the active HslVU complex is a cylindrical four ring structure, composed of  **HslV** , a two ring dodecamer, sandwiched between  **HslU**  rings.  Ref: 9165063 Biol Chem< MedlineTA>Biol Chem, 2000
- A feature in the Haemophilus influenzae HslVU structure, insertion of the C termini of  **HslU**  into pockets in  **HslV** , was not seen in all other structures of the enzyme.  Ref: 12032294 Proc Natl Acad Sci U S A, 2002
- BACKGROUND The bacterial heat shock locus  **HslU**  ATPase and  **HslV**  peptidase together form an ATP dependent HslVU protease.  Ref: 11250202 Structure Camb < MedlineTA>Structure Camb , 2002
- PCR walking on genomic DNA of Leptospira borgpetersenii serovar hardjobovis has identified a genetic locus comprising two heat shock genes  **hslV**  and  **hslU** .  Ref: 11685516 Curr Microbiol, 2001
- Mutational studies on  **HslU**  and its docking mode with  **HslV** .  Ref: 11114186 Proc Natl Acad Sci U S A, 2000
- It is proposed that when overproduced, the  **HslV**   **HslU**  protease complex can degrade SulA which is an endogenous inhibitor of the essential cell division protein FtsZ.  Ref: 9315728 FEBS Lett, 1997
- **HslV**  and  **HslU**  display sixfold symmetry, ruling out mechanisms of protease activation that require a symmetry mismatch between the two components.  Ref: 10693812 Nature, 2000
- Protease  **HslV**  and ATPase  **HslU**  form an ATP dependent protease in bacteria.  Ref: 11722168 J Struct Biol, 2001
- only the originally reported quaternary arrangement of  **HslV**  and  **HslU**  particles is consistent with the experimental data.  Ref: 11722168 J Struct Biol, 2001
- One such locus was identified and shown to carry the recently sequenced  **hslV**   **hslU**  clpQ clpY operon.  Ref: 9003766 EMBO J, 1996
- It consists of  **HslV** , the protease, and  **HslU** , the ATPase and chaperone.  Ref: 12646382 Biophys Chem, 2003
- Here we report the crystal structures of free  **HslU**  and an 82 00 relative molecular mass complex of  **HslU**  and  **HslV**  the first structure of a complete set of components of an ATP dependent protease.  Ref: 10693812 Nature, 2000
- Structure and Reactivity of an Asymmetric Complex between  **HslV**  and I domain Deleted  **HslU** , a Prokaryotic Homolog of the Eukaryotic Proteasome.  Ref: 12823960 J Mol Biol, 2003
- HslVU is a new Escherichia coli ATP dependent protease composed of two multimeric complexes the  **HslU**  ATPase and the  **HslV**  peptidase.  Ref: 9261150 J Biol Chem, 1997
- Hs1VU in E. coli is a new type of ATP dependent protease composed of two heat shock proteins, the  **HslU**  ATPase and the  **HslV**  peptidase related to certain beta type subunits of the 20S proteasome.  Ref: 8954932 Biochem Biophys Res Commun, 1996
- Eubacterial  **HslV**  and  **HslU**  subunits homologs in primordial eukaryotes.  Ref: 12446803 Mol Biol Evol, 2002
- The quaternary arrangement of  **HslU**  and  **HslV**  in a cocrystal a response to Wang, Yale.  Ref: 11722168 J Struct Biol, 2001
- This is the first molecular study on  **hslV**  and  **hslU**  in a Leptospira species.  Ref: 11685516 Curr Microbiol, 2001
- Thus, ATP hydrolysis accelerates the association of  **HslU**  and  **HslV** , which occurs slowly with the nonhydrolyzable analog.  Ref: 9261150 J Biol Chem, 1997
- Crystal structure of HslUV complexed with a vinyl sulfone inhibitor corroboration of a proposed mechanism of allosteric activation of  **HslV**  by  **HslU** .  Ref: 12054822 J Mol Biol, 2002
- an asymmetric  **HslU**  DeltaI 6  **HslV**  12 complex has been crystallized.  Ref: 12823960 J Mol Biol, 2003
- Crystal structures show that  **HslU**  forms a hexamer with a pore at one end and  **HslV**  forms a dodecamer with translocation pores at both ends of two back to back stacked hexameric rings.  Ref: 11469873 J Struct Biol, 2001
- High similarities > or =57.5% with the prototypical  **HslV**  and  **HslU**  from Escherichia coli and conservation of residues essential for biochemical activity suggested that a functional HslVU complex is present in trypanosomatid protozoa.  Ref: 12446803 Mol Biol Evol, 2002
- By itself,  **HslV**  could slowly hydrolyze these polypeptides, but its activity was stimulated 20 fold by  **HslU**  in the presence of ATP.  Ref: 9288941 Eur J Biochem, 1997
- The structure of the N termini of  **HslV**  and  **HslU**  further suggested mitochondrial localization.  Ref: 12446803 Mol Biol Evol, 2002
- Moreover, deletion of 7 amino acids from the C terminus prevented the ability of  **HslU**  to form an HslVU complex with  **HslV** .  Ref: 12011053 J Biol Chem, 2002
- In the case of  **HslU** , the structure was derived from  **HslU**   **HslV**  cocrystals, combining phase information from MAD and the previously determined  **HslV**  model.  Ref: 11722168 J Struct Biol, 2001
- 125I NIP L3VS covalently modifies the  **HslV**  subunit of the Escherichia coli protease complex  **HslV**   **HslU** , a reaction that requires ATP, and supports a catalytic mechanism shared with that of the eukaryotic proteasome.  Ref: 9192616 Proc Natl Acad Sci U S A, 1997
- To define the role of the Cys residues in the ATP dependent HslVU protease, mutagenesis was performed to replace either Cys261 or Cys287 in  **HslU**  with Val and Cys159 in  **HslV**  with Ser or Ala.  Ref: 9722513 J Biol Chem, 1998
- Maximal proteolytic activity was obtained with a 1 2 molar ratio of  **HslV**  a 250 kDa complex to  **HslU**  a 450 kDa complex.  Ref: 9288941 Eur J Biochem, 1997
- We show that, in vivo,  **HslV**  and  **HslU**  interact and participate in the degradation of abnormal puromycylpolypeptides.  Ref: 9003766 EMBO J, 1996
- EM analysis revealed that  **HslV**  and  **HslU**  are both ring shaped particles.  Ref: 9165063 Biol Chem< MedlineTA>Biol Chem, 1996
- A corrected quaternary arrangement of the peptidase  **HslV**  and atpase  **HslU**  in a cocrystal structure.  Ref: 11469873 J Struct Biol, 2001
- Comparison of  **HslU**  alone with its structure in the HslUV complex reveals several conserved amino acid residues whose side chain interactions differ between the two structures, suggesting that they may be part of a conformational switch that facilitates the release of the  **HslU**  carboxy terminal helices when  **HslV**  binds.  Ref: 11468391 Acta Crystallogr D Biol Crystallogr, 2001
- In HslVU complex, the  **HslU**  and  **HslV**  central pores are aligned, and the proteolytic active sites are sequestered in an internal chamber of  **HslV** , with access to this chamber restricted to small axial pores.  Ref: 12011053 J Biol Chem, 2002
- Comparison of the structure of the HslUV inhibitor complex with that of  **HslV**  without inhibitor and in the absence of  **HslU**  reveals that backbone interactions would correctly position a substrate for cleavage in the HslUV complex, but not in the  **HslV**  protease alone, corroborating the proposed mechanism of allosteric activation.  Ref: 12054822 J Mol Biol, 2002
- Biochemical evidence suggests that  **HslV**  ClpQ is an efficient peptidase whose activity is enhanced by  **HslU**  CIpY in the presence of ATP.  Ref: 9003766 EMBO J, 1996
- Phylogenetic analysis indicated that  **HslV**  and  **HslU**  from trypanosomatids clustered with eubacterial homologs but did not point to any particular bacterial lineage.  Ref: 12446803 Mol Biol Evol, 2002
- Despite detailed crystal and molecular structure determinations of free  **HslV**  and  **HslU** , the mechanism of ATP dependent peptide and protein hydrolysis remained unclear, mainly because the productive complex of  **HslV**  and  **HslU**  could not be unambiguously identified from the crystal data.  Ref: 11114186 Proc Natl Acad Sci U S A, 2000
- HslVU in Escherichia coli a new two component ATP dependent protease composed of two heat shock proteins, the  **HslU**  ATPase and the  **HslV**  peptidase which is related to proteasome beta type subunits.  Ref: 9288941 Eur J Biochem, 1997
- HslVU in E. coli is a new type of ATP dependent protease consisting of two heat shock proteins the  **HslU**  ATPase and the  **HslV**  peptidase that has two repeated Thr residues at its N terminus, like certain beta type subunit of the 20S proteasomes.  Ref: 9257689 FEBS Lett, 1997
- CodW and CodX, encoded by the cod operon in Bacillus subtilis, display 52% identity in their amino acid sequences to  **HslV**  and  **HslU**  in Escherichia coli, respectively.  Ref: 11179218 EMBO J, 2001
- Reaction in the crystals with a vinyl sulfone inhibitor reveals that the  **HslU**  DeltaI complexed  **HslV**  hexamer is active, while the uncomplexed  **HslV**  hexamer is inactive.  Ref: 12823960 J Mol Biol, 2003
- HslVU is an ATP dependent protease consisting of two multimeric components the  **HslU**  ATPase and the  **HslV**  peptidase.  Ref: 9299555 Biochem Biophys Res Commun, 1997
- HslVU is an ATP dependent protease consisting of two multimeric components, the  **HslU**  ATPase and the  **HslV**  peptidase.  Ref: 10452560 FEBS Lett, 1999
- We showed that genes homologous to eubacterial  **HslV**  ClpQ and  **HslU**  ClpY are present in the genome of trypanosomatid protozoa and are expressed.  Ref: 12446803 Mol Biol Evol, 2002
- **HslU**  ATPase forms a hexameric ring, and  **HslV**  peptidase is a dodecamer consisting of two stacked hexameric rings.  Ref: 12011053 J Biol Chem, 2002
- The ATPase activity of  **HslU**  was stimulated up to 50% by the protein substrates, but not by nonhydrolyzed proteins, and this stimulation further increased 2 3 fold in the presence of  **HslV** .  Ref: 9288941 Eur J Biochem, 1997
- Identification of the  **hslV**  and  **hslU**  genes in a Leptospira species and the high degree of similarity of the encoded proteins to the E. coli homologs suggest that the encoded  **HslV**  and  **HslU**  proteins function as forming an ATP dependent protease complex as in E. coli.  Ref: 11685516 Curr Microbiol, 2001
- Moreover, either mutation abolished both the basal and  **HslU**  activated proteolytic activity of  **HslV**  and its ability to activate the  **HslU**  ATPase and to form the HslVU complex, indicating that Cys159 is essential for the proteolytic activity of  **HslV**  and its interaction with  **HslU** .  Ref: 9722513 J Biol Chem, 1998
- Whereas the structures of the components were confirmed in detail by later structures, the quaternary arrangement of  **HslV**  and  **HslU**  was not reproduced in later crystal forms.  Ref: 11722168 J Struct Biol, 2001
